# GRHL2 Regulation of Growth/Motility Balance in Luminal versus Basal Breast Cancer

**DOI:** 10.3390/ijms24032512

**Published:** 2023-01-28

**Authors:** Zi Wang, Bircan Coban, Chen-Yi Liao, Yao-Jun Chen, Qiuyu Liu, Erik H. J. Danen

**Affiliations:** Leiden Academic Center for Drug Research, Leiden University, Einsteinweg 55, 2333CC Leiden, The Netherlands

**Keywords:** breast cancer, luminal-like, basal-like, GRHL2

## Abstract

The transcription factor Grainyhead-like 2 (GRHL2) is a critical transcription factor for epithelial tissues that has been reported to promote cancer growth in some and suppress aspects of cancer progression in other studies. We investigated its role in different breast cancer subtypes. In breast cancer patients, GRHL2 expression was increased in all subtypes and inversely correlated with overall survival in basal-like breast cancer patients. In a large cell line panel, GRHL2 was expressed in luminal and basal A cells, but low or absent in basal B cells. The intersection of ChIP-Seq analysis in 3 luminal and 3 basal A cell lines identified conserved GRHL2 binding sites for both subtypes. A pathway analysis of ChIP-seq data revealed cell-cell junction regulation and epithelial migration as well as epithelial proliferation, as candidate GRHL2-regulated processes and further analysis of hub genes in these pathways showed similar regulatory networks in both subtypes. However, GRHL2 deletion in a luminal cell line caused cell cycle arrest while this was less prominent in a basal A cell line. Conversely, GRHL2 loss triggered enhanced migration in the basal A cells but failed to do so in the luminal cell line. ChIP-Seq and ChIP-qPCR demonstrated GRHL2 binding to CLDN4 and OVOL2 in both subtypes but not to other GRHL2 targets controlling cell-cell adhesion that were previously identified in other cell types, including CDH1 and ZEB1. Nevertheless, E-cadherin protein expression was decreased upon GRHL2 deletion especially in the luminal line and, in agreement with its selectively enhanced migration, only the basal A cell line showed concomitant induction of vimentin and N-cadherin. To address how the balance between growth reduction and aspects of EMT upon loss of GRHL2 affected in vivo behavior, we used a mouse basal A orthotopic transplantation model in which the GRHL2 gene was silenced. This resulted in reduced primary tumor growth and a reduction in number and size of lung colonies, indicating that growth suppression was the predominant consequence of GRHL2 loss. Altogether, these findings point to largely common but also distinct roles for GRHL2 in luminal and basal breast cancers with respect to growth and motility and indicate that, in agreement with its negative association with patient survival, growth suppression is the dominant response to GRHL2 loss.

## 1. Introduction

Breast cancer is the most prevalent malignancy in females globally. The mortality of patients with breast cancer has decreased, resulting from early diagnosis and the development of therapies [1,2,3]. A considerable proportion of knowledge on breast cancer originates from experiments performed with breast cancer cells that cover the various subtypes of this heterogeneous disease [4]. Breast cancer is divided into luminal (luminal A and luminal B), epidermal growth factor receptor 2-enriched (HER2-enriched), basal (basal A and basal B), claudin-low, and normal-like subtypes based on gene expression profiling [5]. The most common subtype of breast cancer is luminal which originates from luminal cells in the duct [6]. It is characterized by enrichment of genes/proteins associated with the luminal epithelial phenotype (e.g., ESR1, GATA3 and FOXA1) [4,7]. Basal breast cancer is characterized by significant enrichment of basal epithelial cytokeratins, hormone receptor negativity, and a high tumor grade and poor prognosis [8]. Basal breast cancer can be further divided into basal A and basal B subtypes [9]. The basal A subtype is enriched with basal markers such as cytokeratins (e.g., Cytokeratin 4), while basal-B exhibits a mesenchymal or a normal-like phenotype with overexpression of several genes related to tumor invasion and tumor stemness [4].

The Grainyhead (GRH) gene was originally discovered through a mutation that causes slack and fragile cuticles in Drosophila [10]. Loss of function of the GRH results in failure of neural tube closure during embryogenesis [11]. In mammals, members of the highly conserved Grainyhead-like (GRHL) family directly or indirectly regulate transcription of the genes encoding epithelial cell-cell junction proteins in adherens junctions and tight junctions [12,13,14]. In humans, GRHL1, GRHL2, and GRHL3 are identified as GRH homologs that contain an N-terminal transcriptional activation domain, a central CP2 DNA-binding domain, and a C-terminal dimerization domain [15,16]. GRHL2 has been found to serve as a pioneer factor, cooperating with FOXA1 and ER⍺ to regulate gene expression [15,17,18]. A total of >5000 GRHL2 binding sites have been reported in epithelial cells, but there is limited overlap between different tissues and regulation of several GRHL2-responsive genes have been found to be indirect [13,14,19,20,21].

GRHL2 has been implicated in cancer development and progression. GRHL2 has been shown to act as a tumor metastasis suppressor, by opposing epithelial-mesenchymal transition (EMT) through upregulation of epithelial markers or downregulation of mesenchymal markers [20,22,23]. In contrast, GRHL2 is located on chromosome 8q22 which is frequently amplified or overexpressed in many cancers and hence may rather have an oncogenic function [24,25,26,27]. Indeed, in prostate cancer [28], breast cancer [23], lung cancer [29] and ovarian cancer [30] downregulation of GRHL2 has been associated with the inhibition of cell proliferation. Together, this suggests that GRHL2 function may vary depending on the cancer cell context.

In this study, we investigated the role of GRHL2 in different breast cancer subtypes. Our findings show that GRHL2 is absent in basal B breast cancer cells, and it is expressed in luminal breast cancer cells where its depletion causes an arrested proliferation. Further, it is expressed in basal A where its depletion triggers a slow growth/high motility phenotype and in vivo, growth arrest is the dominant response to GRHL2 depletion, in line with its overexpression in breast cancer patients.

## 2. Results

### 2.1. GRHL2 Is Associated with Poor Prognosis but Is Downregulated in Basal B Subtype Breast Cancer

**Figure 1:**
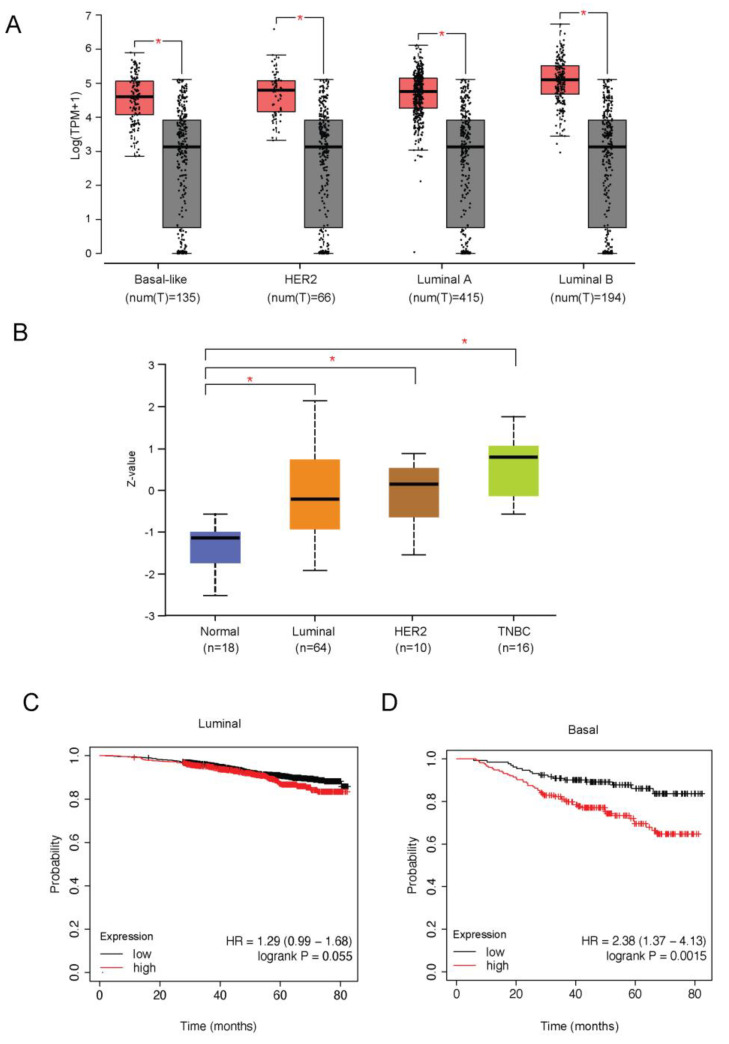
GRHL2 expression in breast cancer. (**A**,**B**) The expression of GRHL2 mRNA (**A**) and protein (**B**) in different subtypes of breast cancer based on analyzing data from TCGA, GTEx, and CPATC databases. TPM, transcripts per million. * Indicates *p* < 0.01. Red and gray blocks in (**A**) represent tumor and normal samples, respectively. Dots show full distribution of all samples in the given group. (**C**,**D**) Association of GRHL2 expression with overall survival based on analyzing data from KM plotter database.

**Figure 2:**
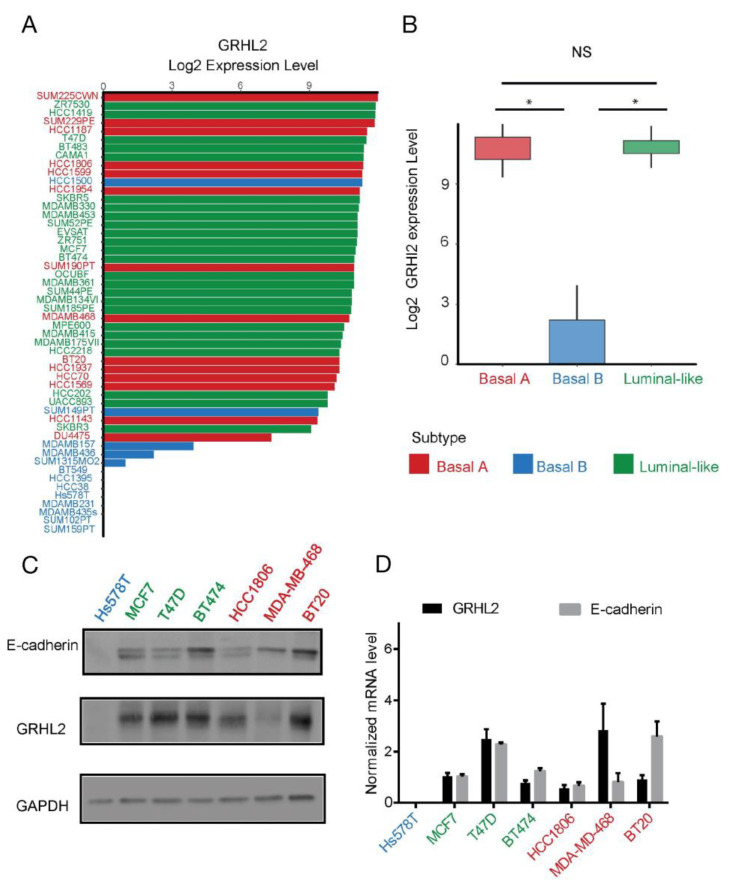
GRHL2 expression in a panel of human breast cancer cell lines representing different subtypes. (**A**,**B**) GRHL2 expression in a panel of >50 human breast cancer cell lines covering luminal, basal A, and basal B subtypes extracted from RNA-seq data. * indicates *p* < 0.05; NS, not significant. (**C**,**D**) Western blot analysis (**C**) and qRT-PCR (**D**) showing loss of GRHL2 and its target gene CDH1 in basal B subtype breast cancer. Color codes refer to (**B**).

In order to evaluate the clinical relevance of GRHL2 in breast cancer, GRHL2 alterations were examined in a series of published cohorts. GRHL2 is located on chromosome 8q22.3, a genomic region that is frequently amplified or overexpressed in many cancers [25,28]. The expression of GRHL2 mRNA and protein was significantly higher in tumor versus normal tissue for all breast cancer subtypes analyzed (Figure 1A,B). No statistically significant association of GRHL2 mRNA expression with overall survival was detected in luminal-like breast cancer (Figure 2C). However, GRHL2 mRNA expression was negatively associated with overall survival in basal-like breast cancer patients (Figure 2D). In agreement with an earlier report exploring a different panel of cell lines [22], using RNA-seq data of 52 human breast cancer cell lines [31] we found that GRHL2 mRNA was low or absent in most breast cancer cell lines representing the basal B subtype and expressed in all luminal and basal A cell lines (Figure 2A,B). Notably, this analysis suggested that HCC1500 and SUM149PT may have been misclassified. Indeed, SUM149PT has been previously classified as basal A or basal B subtype [9,32] and reported to contain different subpopulations, according to expression level of EpCAM and CD49f surface markers [33]. Likewise, HCC1500 cells have been classified as luminal, due to a predominant population of cells that are positive for EpCAM and CD24 [3] or as basal B, owing to an enrichment for gene clusters associated with cancer stem cell and invasive phenotypes [9]. In agreement with the RNA-seq analysis, GRHL2 protein and mRNA were not detectable in Hs578T basal B cells, whereas luminal (MCF7, T47D, and BT474) and basal A cell lines (HCC1806, MDA-MB-468, and BT20) all expressed GRHL2, albeit at different levels (Figure 2C,D). This was correlated with expression of E-cadherin, a cell adhesion receptor previously identified as a target of GRHL2 that is downregulated in cells undergoing EMT [23,34].

### 2.2. Analysis of GRHL2-Occupied Genes in Luminal and Basal A Breast Cancer Cells Points to Overlapping Regulation of Epithelial Proliferation, Cell-Cell Junctions, and Cell Migration

**Figure 3:**
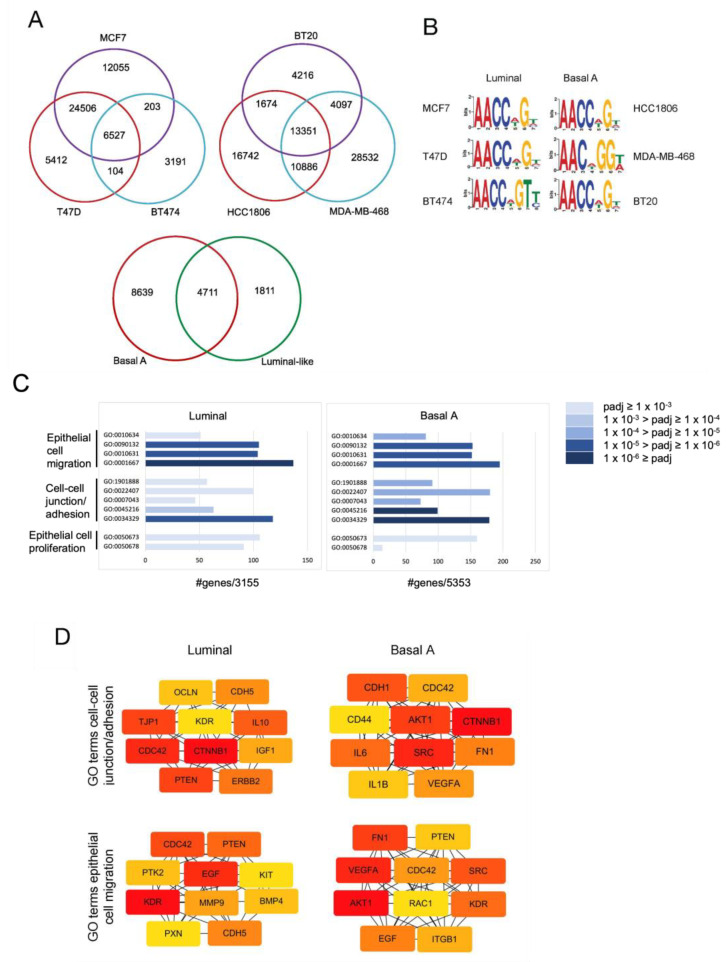
ChIP-seq analysis of GRHL2 target genes in luminal and basal A cells. (**A**) Venn diagrams showing overlap of GRHL2 binding sites among the indicated luminal and basal A cell lines (top panels) and overlap between shared GRHL2 binding sites in luminal and basal A cell lines (bottom panel). (**B**) GRHL2 DNA-binding motifs identified in the indicated cell lines. (**C**) Enriched GO terms associated with the indicated functions for GRHL2-occupied genes shared between all luminal (left panel) or all basal A cell lines (right panel). Color coding according to padj values in the legend. X-axis shows the number of genes involved. (**D**) Hub genes calculated by degree algorithm using Cytoscape (cytoHubba) software from the indicated GO terms for luminal and basal A cells. Color coding from red to yellow indicates the rank of the genes from top to low as assigned by cytoHubba.

We performed ChIP-seq in three human luminal breast cancer cell lines and three human basal A breast cancer cell lines and identified 6527 shared GRHL2 binding sites in all luminal and 13,351 shared GRHL2 binding sites in all basal A cell lines (Figure 3A). Of these, 4711 GRHL2 binding sites were shared between luminal and basal A cells. MEME-ChIP identified a core GRHL2 binding motif matching previously published motifs [13,14,19,21] in each cell line (Figure 3B). Annotation of ChIP-seq data in luminal and basal A cells revealed 3155 and 5353 Ensembl annotations for GRHL2 occupied genes, respectively. These genes were interrogated using the clusterProfiler package in R for GO term enrichment. Several GO terms associated with epithelial proliferation, cell-cell junctions, and cell migration were identified (Figure 3C). We combined GO terms associated with cell-cell junction/adhesion or GO terms associated with epithelial cell migration from Figure 3C and used Cytoscape (cytoHubba) to identify and select hub genes. The resulting hub gene networks were not identical but did show overlap between luminal and basal A cell (Figure 3D), i.e., CDC42 and beta-catenin (CTNNB1) were shared between luminal and basal A in the cell-cell junction/adhesion GO terms. CDC42, PTEN, EGF, and the VEGF receptor KDR (note that the ligand VEGFA was only found in basal A) were shared between luminal and basal A in the epithelial cell migration GO terms. Together, these analyses indicated that gene networks regulated by GRHL2 in luminal and basal A cells show large overlap and may regulate, amongst others, proliferation and cell migration of both subtypes.

### 2.3. Modulation of Proliferation and Migration in Response to GRHL2 Loss in Luminal versus Basal A Breast Cancer Cells

**Figure 4:**
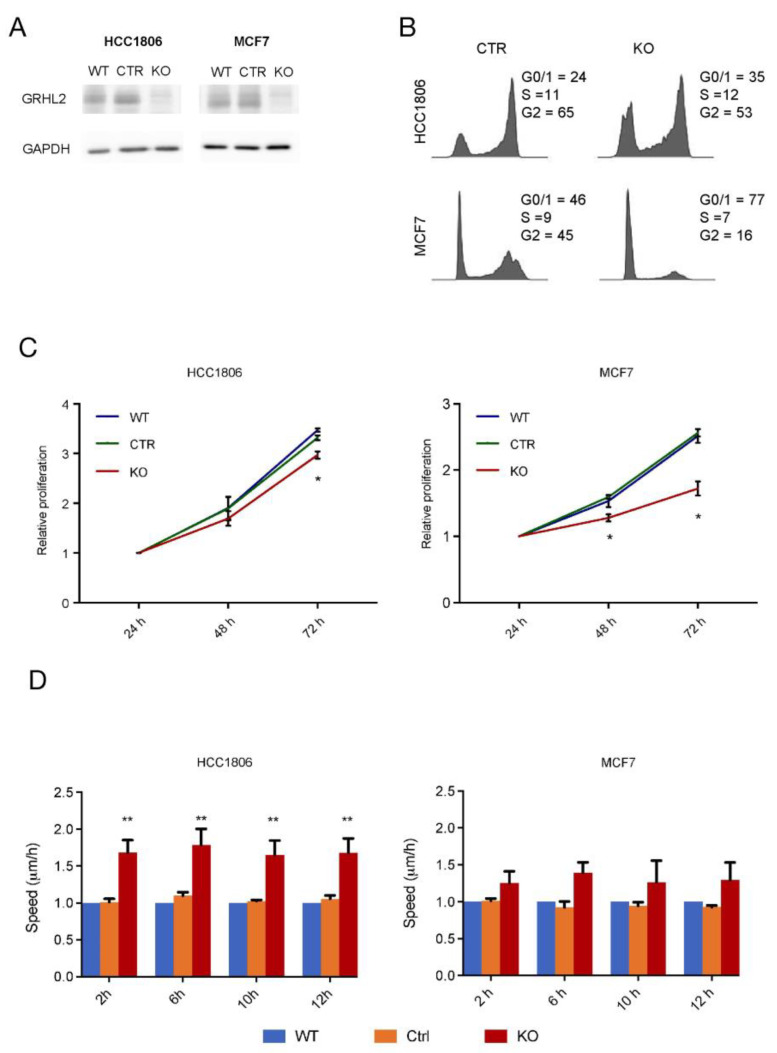
Response to GRHL2 knockout in luminal and basal A cells. (**A**) Western blot analysis showing loss of GRHL2 in KO cells. WT, wild type cells; CTR, sgCTR transduced cells; KO, sgGRHL2 transduced cells. (**B**) FACS profiles with associated quantification of cell cycle phase distribution in sgCTR (CTR) and sgGRHL2 transduced (KO) MCF7 and HCC1806 cells. Representative experiment from 2 and 3 biological replicates is shown for MCF7 and HCC1806, respectively. (**C**) Graphs showing results from SRB assay for wild type (WT) and sgCTR and sgGRHL2 transduced MCF7 and HCC1806 cells for the indicated time periods after 4 days doxycycline. Data are presented as mean ± SEM from 3 biological replicates. Data are statistically analyzed by *t*-test comparing CTR and KO to WT. * indicates *p* < 0.05. (**D**) Analysis of random migration assay showing the average path speed (y-axis) captured at the indicated timepoints during the assay (x-axis) for wild type (WT) and sgCTR and sgGRHL2 transduced MCF7 and HCC1806 cells 10 days post-doxycycline. Data are presented as mean ± SEM from 3 biological replicates relative to WT. Data are statistically analyzed by two-way ANOVA. * indicates *p* < 0.05; ** indicates *p* < 0.01.

GRHL2 has been shown to promote cell survival and proliferation and to suppress EMT in epithelial cells [2,22]. Our ChIP-seq analysis suggested that both aspects could be regulated in luminal as well as basal A breast cancer cells. We studied the response to GRHL2 loss in MCF7 luminal and HCC1806 basal A cells (Figure 4A). Cell cycle analysis showed that a higher percentage of MCF7 cells were in G0/1 compared to HCC1806 cells and loss of GRHL2 resulted in a G0/1 arrest in MCF7 and a less-pronounced shift to G0/1 in HCC1806 cells (Figure 4B). In agreement with the more robust arrest in cell cycle progression observed in MCF7, loss of GRHL2 attenuated cell proliferation of MCF7 cells at 2- and 3-days post-seeding, whereas a smaller, albeit significant decrease in proliferation was observed at 3 days in HCC1806 (Figure 4C). Conversely, when random migration was analyzed, migration speed of HCC1806 cells was enhanced upon GRHL2 depletion, whereas migration of MCF7 was not significantly affected (Figure 4D). Together, these results suggested that GRHL2 loss caused suppression of growth in both subtypes, especially in the luminal cells, that was accompanied by enhanced migration predominantly in the basal A subtype.

### 2.4. Signs of EMT in Response to GRHL2 Loss in Luminal versus Basal A Breast Cancer Cells

**Figure 5:**
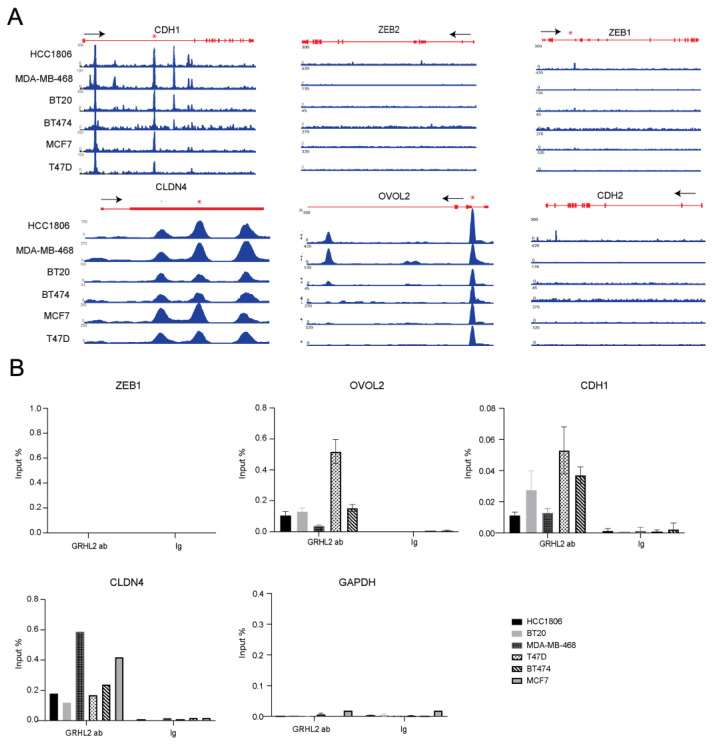
Occupation of EMT related genes by GRHL2. (**A**) ChIP tracks for the indicated genes in luminal and basal A breast cancer cell lines. The track height is scaled from 0 to the indicated number. The locus with its exon/intron structure is presented in red. (**B**) ChIP-qPCR validation of presence and absence of GRHL2 binding sites identified by ChIP-seq. Location of the qPCR primer set in the locus is indicated by * in (**A**). Graphs represent the efficiency of indicated genomic DNA co-precipitation with anti-GRHL2 Ab or IgG control Ab. Signals for IgG control and GRHL2 antibody pulldown samples were normalized to input DNA and are presented as % input with SEM from 3 technical replicates with the exception of CLDN4 due to depletion of input material. Data were statistically analyzed by *t*-test and * indicates *p* < 0.05.

We next asked whether the selective increase in migration speed triggered by GRHL2 loss in basal A but not luminal cells may be related to differential regulation of EMT. Analysis of ChIP tracks in luminal and basal A cell lines for previously reported GRHL2 target genes revealed high similarity amongst all six cell lines (Figure 5A). No interactions of GRHL2 with ZEB1 or ZEB2 were detected in contrast to previous findings in mammary epithelial cells [35]. This was confirmed by ChIP-qPCR using a primer set reported to amplify ZEB1 promoter DNA sequences bound by GRHL2 in human mammary epithelial cells [22] (Figure 5B). We also did not detect GRHL2 peaks associated with CDH2 (encoding N-cadherin, a mesenchymal marker). No promoter binding but multiple intronic GRHL2 peaks were detected in the CDH1 gene that were conserved in all cell lines and ChIP-qPCR confirmed one of these conserved intronic GRHL2 binding sites. Lastly, GRHL2 binding at the promoter regions of CLDN4 and OVOL2 was conserved in all cell lines and validated by ChIP-qPCR.

**Figure 6:**
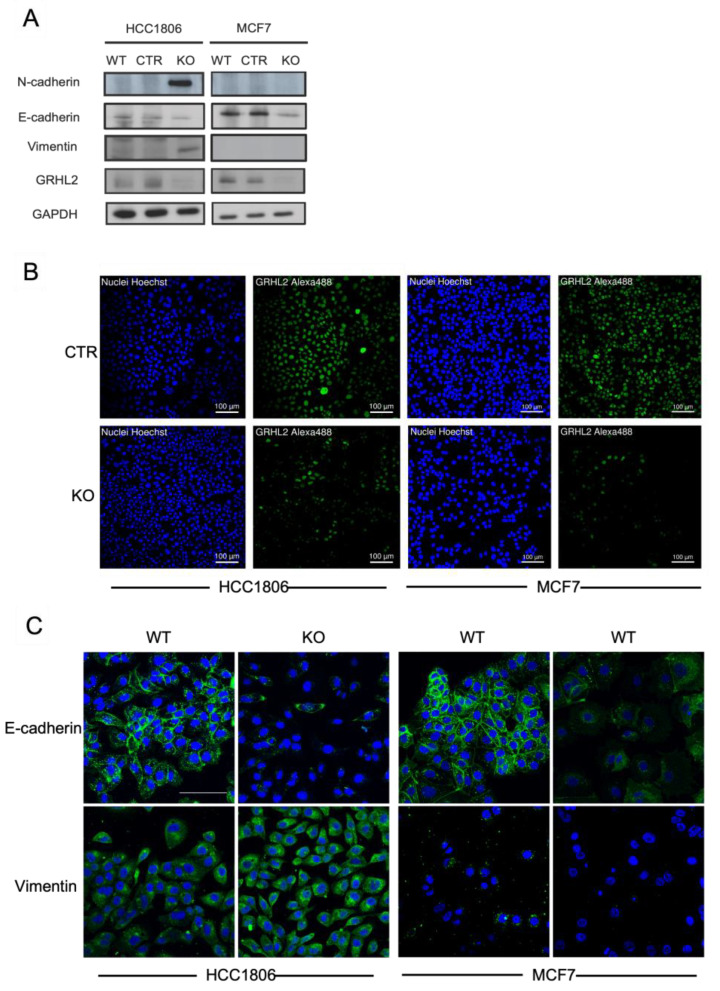
Regulation of EMT related genes by GRHL2. (**A**) Western blot analysis of the indicated proteins in wild type (WT) and sgCTR (CTR) and sgGRHL2 transduced (KO) MCF7 and HCC1806 cells after 10 days doxycycline-induction. (**B**) Immunofluorescence analysis of HOECHST (nuclei, blue) and GRHL2 in MCF7 and HCC1806 cells expressing control sgRNA or GRHL2 sgRNA. (**C**) Immunofluorescence analysis of HOECHST (nuclei, blue), E-cadherin and Vimentin (Alexa-488, green), and F-actin (Rhodamine-Phalloidin, red) in WT, CTR, and KO MCF7 and HCC1806 cells after 10 days doxycycline-induction. Scale bar, 100 µm.

In addition to the direct binding of regulators of EMT, GRHL2 may regulate EMT indirectly. In luminal (MCF7) and basal A cells (HCC1806), GRHL2 knockout, but not control sgRNA, triggered a reduction in E-cadherin protein expression (Figure 6A). However, the induction of mesenchymal markers, Vimentin and N-cadherin, was only observed in HCC1806 cells. These Western blot results were confirmed using confocal immunofluorescence microscopy. E-cadherin was expressed at cell-cell junctions and in the cytoplasm in HCC1806 and MCF7 cells and GRHL2 knockout led to reduced expression (Figure 6B,C). A concomitant gain of Vimentin expression was only observed in HCC1806 cells. These results showed that direct binding of GRHL2 to EMT-related genes is shared between luminal and basal A cells, but GRHL2 depletion triggers several aspects associated with an EMT in HCC1806, whereas only reduction of E-cadherin is observed in MCF7, which may be in agreement with the enhanced migration observed in GRHL2-depleted HCC1806 but not MCF7 cells.

### 2.5. GRHL2 Depletion Leads to Reduced Tumor Growth and Lung Metastasis of 4T1 Basal A Cells

**Figure 7:**
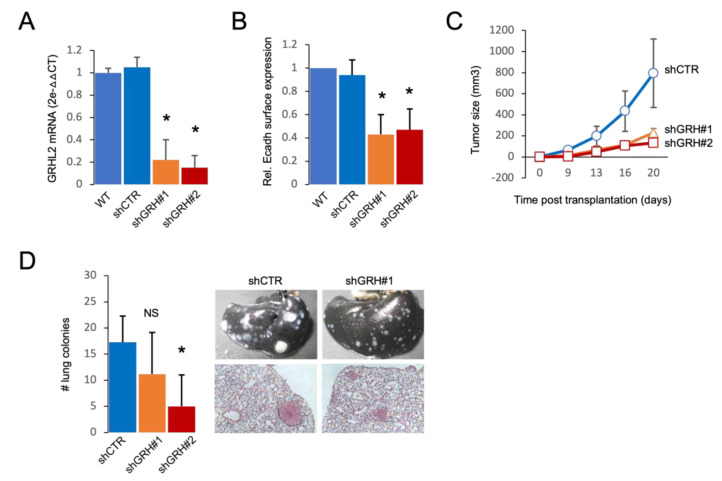
Effect of GRHL2 depletion in 4T1 basal A orthotopic transplantation model. (**A**) RT qPCR analysis of efficiency of GRHL2 depletion in 4T1 cells transduced with a control or 2 GRHL2 shRNA constructs. Mean and SEM of 3 biological replicates is shown. * *p* < 0.05. (**B**) FACS analysis of E cadherin surface expression in 4T1 variants described in A. Mean and SD of 3 experiments is determined and relative MFU (mean fluorescence units) compared to WT is shown. * *p* < 0.05. (**C**) Graph showing mean and SD for analysis of primary tumor growth after orthotopic transplantation of control and GRHL2 shRNA transduced 4T1 cells. At least 12 mice per condition in two experiments were analyzed. (**D**) Graph showing mean and SD for number of detected lung colonies for the experiment as in (**C**). NS, not significant; * *p* < 0.05. Right panel shows representative images of total lungs (top) and hematoxylin/eosin-stained lung sections derived from tumors of 4T1 cells transduced with a control or a GRHL2 shRNA construct.

A shift from an epithelial towards a mesenchymal phenotype contributes to metastasis [36]. We asked whether GRHL2 depletion in basal A cells would attenuate metastasis due to reduced growth potential or promote metastasis due to the EMT shift. For this purpose, we made use of the 4T1 mouse basal A orthotopic transplantation model. Expression of shRNAs targeting GRHL2 led to ~80% reduction in GRHL2 mRNA and caused a decrease in E cadherin expression (Figure 7A,B). Orthotopic tumor growth of shGRHL2 cells was attenuated as compared to growth of shCTR tumors (Figure 7C). Depletion of GRHL2 also reduced lung metastasis with fewer and very small lung colonies (Figure 7D). These results indicated that the dominant outcome of GRHL2 loss in basal A breast cancer cells in the context of tumor growth and metastasis is growth suppression.

## 3. Discussion

The results of this study indicate that the role of GRHL2 in luminal versus basal breast cancer cells is similar but not identical. Shared as well as distinct gene sets are occupied by GRHL2 in these subtypes and the outcome of a reduction in GRHL2 expression may be distinct with luminal cells experiencing a robust growth arrest and basal A cells maintaining a reduced growth potential that is accompanied by enhanced migration. Nevertheless, in agreement with its overexpression and negative correlation with survival in human basal-like breast cancer patients, loss of GRHL2 in basal A cells leads to reduced growth of orthotopic tumors and lung colonies, indicating that growth suppression is the dominant response to GRHL2 loss.

GRHL2 is located on chromosome 8q22 and amplified or overexpressed in several cancer types, including breast cancer [15,37]. In vivo and clinical studies support an oncogenic role of GRHL2 [15,27,28,29,30,38,39]. Our findings corroborate such a role for GRHL2 and demonstrate an association of GRHL2 expression with poor prognosis in breast cancer. Our study using a panel of >50 human breast cancer cell lines confirms and extends an earlier report showing that GRHL2 is downregulated in basal B breast cancer [22]. This is remarkable given its apparent relation to poor prognosis, since triple negative/basal tumors are often aggressive and have a poorer prognosis compared to the ER-positive luminal subtypes [4]. Moreover, basal B cells are enriched in EMT markers that are also associated with aggressiveness [4].

GRHL2 may play a dual role in breast cancer [15,16,23] and a tumor- or metastasis-suppressive function has been related to its ability to suppress EMT, stemness, and invasion in cell line models and clinical samples [22,23,35]. The function of GRHL2 likely is context-dependent and the consequence of GRHL2 loss depends on the cancer type and the stage of cancer progression. Here, we have explored whether luminal and basal A breast cancer cells that are GRHL2 positive and have an epithelial phenotype with E-cadherin-mediated cell-cell contacts, respond similarly to a loss of GRHL2. We find that GRHL2 downregulation negatively impacts growth and positively impacts aspects of EMT/migration in a luminal as well as a basal A cell line, but not to the same extent. Growth inhibition upon loss of GRHL2 is robust in MCF7 but less pronounced in HCC1806 cells. Notably, expression of PCNA and TERT, which has been reported to be epigenetically controlled by GRHL2 [26,40] was attenuated in MCF7 but not HCC1806 cells following GRHL2 depletion, indicating that replicative potential is differentially affected.

As expected, based on earlier reports in other cell types [21,41,42,43], E-cadherin is downregulated in response to GRHL2 knockout in luminal as well as basal A breast cancer cells. Our findings indicate that this response may be indirect. ChIP-seq and ChIP-qPCR detect intronic GRHL2 binding sites in the CDH1 gene but no promoter binding. By contrast, we find that genes encoding the epithelial tight junction protein, CLDN4 and the epithelial zinc finger protein, OVOL2 are subject to GRHL2 promoter binding. We do not detect GRHL2 binding sites in ZEB1 or ZEB2, across all the luminal and basal A breast cancer cell lines we have analyzed, which contrasts with previous findings in other cell types [35]. Hence, the regulation of E-cadherin could involve OVOL2 but not ZEBs in breast cancer cells. Notably, GRHL2 binding to noncoding gene regions, such as the intronic binding sites in CDH1, may participate in long-distance chromatin interactions [21]. Moreover, intronic regions harbor enhancer elements and GRHL2, in some cases together with the ER-alpha transcriptional complex, can act as a pioneer factor [15,16,17,18]. As the absence or presence of promoter or intronic binding sites is shared across the luminal and basal A cells, these interactions do not explain the different response to GRHL2 loss in both subtypes. On the other hand, we observe an induction of mesenchymal markers and enhanced cell migration only in GRHL2-depleted basal A HCC1806 cells and not in GRHL2-depleted MCF7 luminal cells. It has been previously shown that loss of E-cadherin is not sufficient for enhanced cell migration, invasion, and metastasis of breast cancer cells [44,45,46]. The induction of mesenchymal markers, such as N-cadherin and Vimentin that we find to occur only in the HCC1806 cells, may contribute to cell migration. N-cadherin junctions on the cell surface may act as migration tracks [47] and N-cadherin supports the organization of an F-actin network that drives cell migration [48]. Again, the upregulation of N-cadherin in HCC1806 cells appears to be indirect as no GRHL2 bindings sites are detected in the CDH2 gene. Vimentin, a type III intermediate filament protein, is involved in cell adhesion, migration, and signal transduction and emerges in pathologies processes involving epithelial cell migration [49], but overexpression of Vimentin by itself does not enhance cell migration in MCF7 cells [50]. Altogether, our findings and other reports indicate that in order for GRHL2 loss to trigger a shift to more motile behavior, loss of E-cadherin is not sufficient, but a more elaborate transition is required, including loss of epithelial markers and gain of mesenchymal markers.

Despite the induction of EMT and enhanced migratory capacity in basal A breast cancer cells, the ultimate outcome of GRHL2 depletion in the orthotopic tumor growth and metastasis experiment reported here supports an oncogenic role of GRHL2. This is in agreement with earlier studies [28,30,51] and with the fact that GRHL2 is located on chromosome 8q22 which is amplified or overexpressed in several cancer types, including breast cancer [15,37]. Our findings show that GRHL2 represents a candidate therapeutic target for luminal breast cancer. Even though growth of a primary tumor and metastatic colonies derived from basal breast cancer cells is suppressed in response to GRHL2 depletion, the combination of reduced growth with aspects of EMT and enhanced motility does warrant caution with strategies aimed at decreasing expression of GRHL2 in basal breast cancer.

## 4. Materials and Methods

### 4.1. Expression Analysis in Breast Cancer Cohorts and Cell Line Panel

GRHL2 mRNA and protein expression in different subtypes were analyzed using GEPIA2 [52] and UALCAN [53,54], respectively. The KM plotter database was analyzed to evaluate the association of GRHL2 expression with the survival of patients with different subclasses of breast cancer [34,55]. RNA-seq data for a panel of 52 breast cancer lines [35] was used to analyze GRHL2 expression across subtypes (basal A, basal B, and luminal).

### 4.2. Cell Lines

Human breast cancer cell lines (MCF7, T47D, BT474, HCC1806, BT20, MDA-MB-468, Hs578T) were obtained from the American Type Culture Collection. Cells were cultured in RPMI1640 medium with 10% fetal bovine serum, 25 U/mL penicillin, and 25 µg/mL streptomycin in the incubator (37 °C, 5% CO_2_).

For the production of lentiviral particles, VSV, GAG, REV and Cas9 or sgRNA or shRNA plasmids were transfected into HEK293 cells using Polyethylenimine (PEI). After 2 days, lentiviral particles were harvested and filtered. Conditional Cas9 cells were generated by infecting parental cells with lentiviral particles expressing Edit-R Tre3G promotor-driven Cas9 (Dharmacon) and selected by blasticidin. Limited dilution was used to generate Cas9 monoclonal cells. Subsequently, Cas9-monoclonal MCF7 or HCC1806 cells were transduced with U6-gRNA:hPGK-puro-2A-tBFP control non-targeting or GRHL2-specific single guide (sg)RNAs (Sigma) selected by puromycin, and gene deletion was triggered by exposure to doxycycline. Control sgRNA vector was non-targeting. Human GRHL2 sgRNAs were CACCGACAGCAGCCTCTTGTCTCGGT and TAAAACCGAGACAAGAGGCTGCTGTC. For shRNA transduction of 4T1 cells, cells were transduced using lentiviral shRNA vectors (LentiExpress; Sigma-Aldrich, Zwijndrecht NL) according to the manufacturer’s procedures and selected in medium containing puromycin (2 mg/mL). Control shRNA vector targeted enhanced green fluorescent protein. Mouse GRHL2 shRNAs were CGGAGAAATTTCGGAGTACTTCTC and CGTCCTTGTTAAGCGGATGTTCTC.

### 4.3. Western Blot

Cells were lysed by radioimmunoprecipitation (RIPA) buffer (150 mM NaCl, 1% Triton X-100, 0.5% sodium deoxycholate and 0.1% Tris and 1% protease cocktail inhibitor (Sigma-Aldrich, Zwijndrecht NL; P8340). Then, cell lysates were sonicated and protein concentration was determined by bicinchoninic acid assay (BCA). Cell lysates were mixed with protein loading buffer. Subsequently, proteins were separated by SDS-PAGE gel and transferred to methanol-activated polyvinylidene difluoride (PVDF) (Milipore, Amsterdam NL) membrane. Membranes were blocked with 5% bovine serum albumin (BSA; Sigma-Aldrich, Zwijndrecht NL) for 1 h at room temperature (RT). Membranes were stained with primary antibody overnight at 4 °C and HRP-conjugated secondary antibodies for half hour at room temperature (RT). After staining with Prime ECL Detection Reagent (GE Healthcare Life science, Hoevelaken NL), chemoluminescence was detected by an Amersham Imager 600 (GE Healthcare Life science, Hoevelaken NL). The following antibodies were used: GRHL2 (Atlas-Antibodies, hpa004820; Bio-connect BV, Huissen NL), GAPDH (SantaCruz, sc-32233; Bio-connect BV, Huissen NL), Vimentin (Abcam, ab8069; Abcam BV, Amsterdam NL), N-cadherin (BD Biosciences, Vianen NL, 610920), E-cadherin (Abcam, ab76055; Abcam BV, Amsterdam NL), Peroxidase AffiniPure Goat Anti-Rabbit IgG (Jackson ImmunoResearch, 111-035-003; SANBIO BV, Uden NL), and Peroxidase AffiniPure Goat Anti-Mouse IgG (Jackson ImmunoResearch, 115-035-003; SANBIO BV, Uden NL).

### 4.4. Realtime Quantitative PCR (RT-qPCR)

Total RNA was isolated using RNeasy Plus Mini Kit (Qiagen, Venlo NL). A total of 500 ng RNA was reverse transcribed into cDNA using the RevertAid H Minus First Strand cDNA Synthesis Kit (Thermo Fisher Scientific, Leiden NL). The cDNA was mixed with SYBR green master mix (Thermo Fisher Scientific, Leiden NL) for qPCR. RT-qPCR data were collected and analyzed using 2−ΔΔCt method. RT-qPCR primers included GRHL2 forward: ggcagtgtccttgttaaacgg/reverse atcgtcagtctccttcctcacg; CDH1 forward: agagcttgtcattgagcctgg/reverse: ccacggatcttgtgtcagaaac; GAPDH forward: ccatggggaaggtgaaggtc/reverse agttaaaagcagccctggtga.

### 4.5. ChIP-Seq and ChIP-qPCR

Cells were grown in serum containing RPMI-1640 Complete Medium. Cross-linking was performed by 1% formaldehyde for 10 min at room temperature (RT). Then 1 M glycine (141 µL of 1 M glycine for 1 mL of medium) was used to quench for 5 min at RT. Cells were washed twice with ice-cold PBS containing 5 µL/mL phenylmethylsulfonyl fluoride (PMSF). Cells were harvested by centrifugation (2095 g for 5 min at 4 °C) and lysed with NP40 buffer (150 mM NaCl, 50 mM Tris-HCl, 5mM EDTA, 0.5% NP40, 1% Triton X-100) containing 0.1% SDS, 0.5% sodium deoxycholate, and protease inhibitor cocktail (EDTA-free Protease Inhibitor Cocktail, Sigma, Zwijndrecht NL). Chromatin was sonicated to an average size of 300 bp and GRHL2-bound chromatin fragments were immunoprecipitated with anti-GRHL2 antibody (Sigma, Zwijndrecht NL; HPA004820). Precipitates were washed by NP buffer, low salt (0.1% SDS, 1% Triton X-100, 2 mM EDTA, 20 mM Tris-HCl (pH 8.1), 150 mM NaCl), high salt (0.1% SDS, 1% Triton X-100, 2 mM EDTA, 20 mM Tris-HCl (pH 8.1), 500 mM NaCl) and LiCl buffer (0.25 M LiCl, 1% NP40, 1% deoxycholate, 1 mM EDTA, 10 mM Tris-HCl (pH 8.1)). Chromatin was de-crosslinked by 1% SDS at 65 °C and DNA was purified by Phenol:Chloroform:Isoamyl Alcohol (PCI) and then diluted in TE buffer. Library preparation and paired-end (151 bp) sequencing were performed by GenomeScan (Leiden NL). MCF7, T47D, BT474, HCC1806, MDA-MB-468, and BT20 had 87393758, 84633440, 82080866, 89366122, 114657768, 62258090 paired-end reads, respectively. ChIP-seq data are available at the UCSC Genome Browser [https://genome.ucsc.edu/s/hwuRadboudumc/ZWang (accessed on 18 June 2020)].

For ChIP-qPCR, the following primers were used: control (an intergenic region upstream of the GAPDH locus): forward atgggtgccactggggatct/reverse tgccaaagcctaggggaaga; CLDN4: forward gtgacctcagcatgggctttga/reverse ctcctcctgaccagtttctctg; ZEB1 promoter: forward gccgccgagcctccaacttt/reverse tgctagggaccgggcggttt; OVOL2 exon: forward ccttaaatcgcgagtgagacc/reverse gtagcgagcttgttgacacc; CDH1 intron: forward gtatgaacggcaagcctctg/reverse caagggagccaggaagagaa. ChIP-qPCR data were analyzed using the 2−ΔΔCt method.

### 4.6. ChIP-Seq Analysis

Less than 5% of adapter sequences were present, and the mean per base sequence quality was >30, indicating high quality reads and no requirement for adapter-trimming. Paired-end reads were mapped to the human reference genome (hg38) using BWA-MEM [56] with default parameters. Mapping of total reads to the human genome for MCF7, T47D, BT474, HCC1806, MDA-MB-468, and BT20 was 42915, 36183, 10054,42554, 56906, 23486, respectively. Phred quality score (Q score) was used to measure base calling accuracy [57] and reads with low mapping quality (≤Q30) were filtered out. MACS version 2.1.0 [58] was used for peak calling by default settings. The q value was adjusted to 0.1 for BT474 cell line to avoid loss of peaks. The annotatePeaks and MergePeaks functions from HOMER [59] were used to annotate and overlap peaks, respectively. ChIPseeker was used for the analysis of ChIP-seq peaks coverage plot and the density profile of GRHL2 binding sites [60]. Motif analysis was performed using ChIP-seq peaks with high scores by the MEME-ChIP program with default settings. ChIP-seq data was visualized by the UCSC genome browser.

The clusterProfiler package in R was used for GO enrichment analysis using the Kyoto Encyclopedia of Genes and Genomes (KEGG) database [61]. For this purpose, gene symbols of annotated ChIP-seq data were converted to Ensembl gene annotations. For the luminal and basal A subtypes 3155 and 5353 Ensembl annotations were analyzed, respectively. Protein-protein interaction (PPI) networks were analyzed using STRING database (https://string-db.org/ (accessed on 7 October 2022)). The cytoHubba plugin in Cytoscape software (version 3.7.2) was used to identify hub genes and their networks. The “Degree” algorithm was used to select the top genes in Cytoscape.

### 4.7. Flow Cytometry

Cell cycle analysis was performed with a Click-iT EdU Flow Cytometry Kit (Invitrogen; Thermo Fisher Scientific, Leiden NL). Cells were cultured with 50 µm 5-ethynyl-2-deoxyuridine (EdU) for 4 h and fixed and stained according to the manufacturers protocol for analysis on a BD FACS Canto II.

### 4.8. Sulforhodamine B (SRB) Assay

Cell proliferation rate was measured by SRB assay. Cells were seeded into 96 well plates. At indicated time points, cells were fixed with 50% trichloroacetic acid (TCA; Sigma-Aldrich, Zwijndrecht NL) for 1 h at 4 °C and then plates were washed with demineralized water four times and air-dried at RT. Subsequently, 0.4% SRB (60 µL/well) was added and kept for at least 2 h at RT. The plates were washed five times with 1% acetic acid and air-dried. 10 mM (150 µL/well) Tris was added and kept for a half-hour at RT with gentle shaking. The absorbance value was measured by a plate-reader Fluostar OPTIMA.

### 4.9. Migration Assay

A total of 96 well-plates were coated with collagen (50 µL/well, 20 µg/mL) for 1 h at 37 °C and washed with PBS. Cells were seeded into the coated 96 well plates at the density of 8000 cells/well overnight and stained with Hoechst (Thermo Fisher, Leiden NL; 33242) diluted 1:7500 for 45 min. Images were taken every 5 min on a Nikon TE confocal microscope; NIKON Europe BV, Leiden NL for 12 h, at two positions per well. Tracks were analyzed using NIS Elements software and migration speed was calculated for 30 cells in each condition, tracked at 25 different timepoints.

### 4.10. Immunofluorescence

Cells were fixed with 2% formaldehyde for 15 min under slow rotation, permeabilized with 1% Triton in Phosphate buffered saline (PBS) for 10 min, and then stained with primary antibodies and secondary antibodies. The following antibodies and stains were used: vimentin (Abcam, ab8069; Abcam BV, Amsterdam NL); E-cadherin (Abcam, ab76055; Abcam BV, Amsterdam NL); GRHL2 (Atlas-Antibodies, hpa004820); Hoechst (Abcam 33258; Abcam BV, Amsterdam NL); Goat anti-Mouse IgG (H + L) Cross-Adsorbed Secondary Antibody-Alexa Fluor 488 (Thermo Fisher, Leiden NL; A-11001); Rhodamine-Phalloidin (Thermo Fisher, Leiden NL; R415).

### 4.11. Animal Studies

Rag2−/−; gc−/− mice were housed in individually ventilated cages under sterile conditions. Housing and experiments were performed according to the Dutch guidelines for the care and use of laboratory animals. Sterilized food and water were provided ad libitum. Tumor cells (1 × 105) in 0.1 mL of phosphate-buffered saline were injected into the fat pad of 8- to 12-week-old female mice. The size of the primary tumors was measured using calipers. After 3 to 4 weeks, animals were anesthetized with pentobarbital. Lungs were excised and left lungs were fixed in 4% paraformaldehyde for hematoxylin and eosin staining. To quantify lung metastases, right lungs were injected with ink solution, destained in water, and fixed in Feketes (4.3% (*v*/*v*) acetic acid, 0.35% (*v*/*v*) formaldehyde in 70% ethanol).

### 4.12. Statistical Analyses

Statistical analyses were performed by GraphPad Prism 8. Details are further described in the figure legends.

## Data Availability

Chip-seq data supporting the results of this article are available at the UCSC Genome Browser [https://genome.ucsc.edu/s/hwuRadboudumc/ZWang (accessed on 18 June 2020)].
